# Midfacial Reconstruction – A Systematic Review

**DOI:** 10.3889/oamjms.2016.067

**Published:** 2016-06-17

**Authors:** Aala Emara, Adel Abou ElFetouh, Maha Hakam, Basma Mostafa

**Affiliations:** 1 *Oral Maxillofacial Department, Faculty of Dentistry, Cairo University, Cairo, Egypt*; 2 *Surgery and Oral Medicine Department, Oral and Dental Research Division, National Research Centre, Cairo, Egypt*

**Keywords:** Midface, reconstruction, oncologic defect, maxillectomy, Maxilla

## Abstract

**AIM:**

Different lesions affecting the midfacial regions require surgical reconstruction. The aim of this study was to assess the different methods used in midfacial reconstruction after maxillectomy procedures. The various reported surgical reconstructive techniques focusing on the esthetic and functional outcomes are to be reviewed in this article.

**MATERIAL AND METHODS:**

A thorough PUBMED and hand-search of journals of relevance was performed on related terms and yielded 772 titles of which 45 abstracts were selected and obtained as full articles for further evaluation while the rest were excluded by title/abstract. According to the inclusion criteria; 14 of these studies were used to complete this article.

**RESULTS:**

In this review we showed that fibular and radial vascularized grafts were the most commonly reported methods in literature with a few other options. Computer aided design and surgical planning has been also reviewed and seems to be a rapidly evolving option for maxillofacial reconstruction. Lack of RCTs (randomized controlled trials) and large scale case series was noticed in this review making the evidence of poor quality.

**CONCLUSION:**

Methods of evaluation of reconstruction options mainly qualitative and subjective made the evaluation of the techniques in this review difficult.

## Introduction

Benign and malignant lesions commonly affecting the maxillary/midface regions require surgical ablation. The surgical removal of such lesions leaves behind a defect of esthetic and functional components. The esthetic component of the defect is caused by the loss of facial support of the soft tissues related to the region especially the cheek prominence resulting in facial asymmetry. In cases where the defect extended to the orbital region causing orbital exenteration the esthetic disfigurement is paramount. The functional problems are caused by the loss of the maxillary alveolar process with subsequent loss of the masticatory function. Moreover, the speech and cosmetic problems they cause; make their reconstruction very difficult [1].

Another functional issue encountered is the oro-nasal/antral communication which occurs in cases of lesions perforating the antral / nasal floor. Palatal defects may be covered by acrylic obturators and sparing the patient the sequelae of oro-antral communication [2]. Obturators have historically been the sole reconstructive option till the introduction of microvascular surgical techniques [3]. Microvascular surgery is widely used to reconstruct maxillary-midface defects overcoming the patient’s dissatisfaction with obturators’ drawbacks and donor site selection [4]. Microvascular reconstruction provides better esthetics, mastication and speech than obturators.

On the other hand, microvascular procedures are complex procedures requiring special training. The donor site needed to harvest the graft adds to the surgical morbidity, increases the intraoperative time and may require more than one surgical reconstructive procedure [5, 6]. The microsurgical techniques introduced are still being updated and researched greatly; there still remains its complex nature and the complications accompanied with it [3].

The various surgical reconstructive techniques used focusing on the esthetic and functional outcomes are to be reviewed in this systematic review.

## Materials and Methods

This review aimed to study the different reported techniques of midfacial reconstruction.

### Search strategy

A search in MEDLINE (Pub Med) was performed using the following search query:

- #1 midface
- #2 rehabilitation OR reconstruction.

A hand search of international journals in the scope of maxillofacial surgery was also performed to identify any skipped relevant articles (British journal of oral and maxillofacial surgery (OMFS), International journal of OMFS, Journal of OMFS, Journal of craniofacial surgery, Journal of craniomaxillofacial surgery, Journal of plastic and reconstructive surgery, Journal of aesthetic plastic surgery, Journal of clinical oral investigation, Journal of clinical otorhinolaryngeology, Journal of craniomaxillofacial trauma and reconstruction, Head & neck journal, Annals of plastic surgery, Journal of prosthetic dentistry, Journal of oral surgery-pathology-radiology-endodontics).

### Study selection

The results of this search yielded 772 titles that were screened by the authors. Forty five of the resulting titles were chosen for abstract evaluation. Clinical trials reporting different techniques of maxillary reconstruction of pathologic defects were selected and after screening 29 articles were obtained as full articles and studied thoroughly and those not fulfilling the inclusion criteria were excluded.

**Figure 1:**
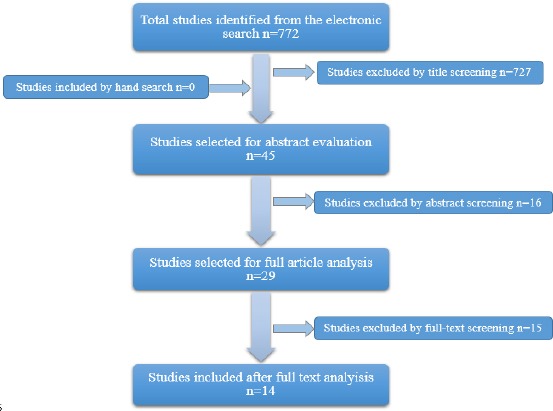
Study selection process

The 14 publications fulfilling the inclusion criteria were selected to perform this review. Author disagreements were negotiated till satisfactory results reached (Figure 1).

Clinical trials reporting different techniques of maxillary reconstruction of pathologic defects were selected and after screening at each level studies were excluded according to the following exclusion criteria.

### Exclusion criteria

- Traumatic fractures of the face (non-pathologic defects)
- Cosmetic / plastic surgeries
- Animal studies
- Studies evaluating surgical approach rather than reconstruction technique
- Soft tissue defects without a bony component
- In languages other than English
- Non clinical trials / review articles

### Data Collection

Data collection forms were custom designed for the selected articles including the following items:

- Authors & study date
- Study type
- Number of patients
- Age of patients
- Treatment provided
- Follow up duration
- Patient satisfaction
- Histopathological assessment

The selected articles were studied according to the method of reconstruction used and outcome accomplished. The different methods of reconstruction reported included; iliac grafts, fibular grafts, radial forearm free flap, scapular, calvarial, computer guided procedures, distraction ostoegenesis or a combination of two of these techniques together.

### Critical appraisal

Risk of bias was assessed according to study design, randomized selection, specification of the inclusion/exclusion criteria, reporting of lost follow-up and complications, objective evaluation and statistical analysis of the results.

## Results

Of the 14 articles selected none were randomized controlled trial; the study design was either retrospective (6 studies), case report (5 studies) or case series (3 studies) and so no meta- analysis was possible and the results will be presented in a descriptive manner. Data was collected from the selected articles in customized forms and tabulated (Table 1) and the risk of bias assessment presented in Table 2.

**Table 1:** list of selected articles

Study – publication, yr	Study design	No. of pts	Mean age (Year)	TTT provided	Followup mean (months)	Histopathologic report	Results (patient satisfaction)
Mueller et al. 2014	Retro.	10	65	-RFFF 3 -Fibular 1 -RFFF + ALT 1 -Iliac +ALT 1	43	SCC Adenoid CC BCC AB	5/10 wore prosthesis 3/10 back to social life
Moreno et al. 2010	Retro.	18	50.8	-Fibular 11 -Fibula +ALT 3 -RFFF 2 -Lateral arm 1 -Serratus composite 1	27.3	N	Unrestricted diet 55% Excellent speech 47.5%
Mertens et al. 2013	Case report	1	50	-Scapular +pt. specific implant	48	Undiff. Pl. S.	Good esthetics after corrective surgery
Echo et al. 2013	Case report	1	52	-MEDPOR implant	9	Cellular myoma	Acceptable esthetics
Dediol et al. 2013	Case series	7	57	-Titanium mesh + LD 1 + ALT 4 + Fibular 1	15	SCC SAC Periosteal sarcoma	Average facial appearance grade = Excellent
Shaw et al. 2009	Case report	1	62	-Ost.my.c. DCIAP flap	1	Antral SCC	Esthetics = Good
Andrades et al. 2008	Retro.	23	67	-Ost.c. RFFF	11	SCC Sarcoma Melanoma	Esthetics = Good
Chepeha et al. 2005	Case series	7	50	-Ost.c. RFFF	27.9	SCC Spindle CC Hemangio.p.c Sarcoma AB Ad. CC	Esthetics = Very good
Bidros et al. 2005	Case report	2	27.5	-TDAPSOC flap	8	Epitheliod s. Adenoid CC	N
Parhiscar et al. 2002	Retro.	2	50	-TOF flap	27	SCC Haemangioma	N
Futran et al. 2002	Retro.	27	59	-Ost.C. Fibular	26	N	Diet:14 normal, 13 soft only; Speech: all good Cosmetics: 14 excellent, 8 good, 4 fair, 1 poor with obturator
Pollice et al. 1988	Case series	6	59.5	-Calvarial +TPF	6.5	AB Ad. CC SCC Met.Thy.C.	Good 1 with moderate deformity
Schmelzem et al. 1998	Retro.	8	47.75	-Scapular	N	Osteosarcoma SCC Oss fibroma	2^nd^ correctional surgery needed in 5 cases
He et al. 2009	Case report	1	21	-3D fibula	6	Osteosarcoma	Good esthetics and function

AB: Ameloblastoma, Ad. CC: Adenoid cystic carcinoma, ALT: anterolateral thigh, BCC: Basal Cell Carcinoma, Met.Thy.C.: Metastatic thyroid carcinoma RFFF: radial forearm flap, LD: Latissmus Dorsi, DCIAP: Deep circumflex iliac artery perforator, OSS: osseus, Ost.my.c: osteomyocutaneous flap, pt: patient, SAC: Sinoantral carcinoma, SCCC: Squamous Cell Carcinoma TOF: Temporoparietal osteofascial, TDAPSOC: Thoracodorsal Artery Perforator-Scapular Osteocutaneous, TPF: Temporoparietal fascial, Retro: retrospective, Undiff Pl. S.: undifferentiated pleomorphic sarcoma.

**Table 2:** Risk of bias assessment for the selected articles

Study	Type of study	Inclusion /exclusion criteria	Selection randomization	Reported loss to follow-up	Reported complications	Objective evaluation	Statistical analysis	Risk of bias
Mueller et al. 2014	Retro.	No	No	No	Yes	No	No	High
Moreno et al. 2010	Retro.	Yes	No	N.I.	Yes	Yes	Yes	Moderate
Mertens et al. 2013	Case report	No	No	No	Yes	No	No	High
Echo et al. 2013	Case report	No	No	No	No	No	No	High
Dediol et al. 2013	Case series	No	No	Yes	Yes	No	No	Moderate
Shaw et al. 2009	Case report	No	No	No	No	No	No	High
Andrades et al. 2008	Retro.	No	No	Yes	Yes	No	No	High
Chepeha et al. 2005	Case series	Yes	No	No	Yes	Yes	Yes	Moderate
Bidros et al. 2005	Case report	No	No	No	Yes	No	No	High
Parhiscar et al. 2002	Retro.	No	No	No	Yes	No	No	High
Futran et al. 2002	Retro.	No	No	No	Yes	No	No	High
Pollice et al. 1988	Case series	Yes	No	No	No	No	No	High
Schmelzem et al. 1998	Retro.	Yes	No	No	Yes	No	No	High
He et al. 2009	Case report	No	No	No	No	No	No	High

The articles were published in the period from 1998 to 2015. The outcome in all of them was a subjective assessment of esthetic and functional patient satisfaction and so no numerical evaluation of the different reconstruction techniques was possible. Histopathologic assessment of the lesions in most cases reported a malignant pathology; and in some cases adjunctive chemotherapy and/or radiotherapy was necessary. Risk of bias in all selected articles was substantially high due to the study design which was either retrospective studies or prospective non-controlled case series/reports. This literature review will therefore only present the studies narrative without any meta-analysis.

### Fibular graft

A total of 55 cases of reconstruction using fibular grafts were found in the search of which 3 were associated with an anterolateral thigh soft tissue flap. Moreno et al. reported the use of an osteocutaneous fibular graft in 11 patients with total loss of the graft in one patient; and partial flap loss of less than 10 % in 2 cases A combination of an ALT and an osteocutaneous fibular graft was reported in 3 of these cases with reported uneventful healing and successful grafting [7]. An osteomyocutaneous fibular graft was used to reconstruct a midface defect using a 3D simulation technique reported as a case report. The patient’s preoperative computed tomography (CT) scans were imported and a virtual osteotomies and graft harvesting performed. The fibular graft to be harvested was adapted virtually as well to reach the best esthetic result preoperatively and to reduce intraoperative time. A 3D resin model of the final reconstructed midface was printed to aid in intraoperative orientation. The authors reported patient’s satisfaction with the function and esthetics; dental rehabilitation was provided 6 months postoperatively [8].

The patients reported excellent (14/27), good (8/27), fair (4/27) and poor (1/27) esthetics. Regarding function, the study reported regaining of normal diet in 14 patients while 13 others only followed a soft diet regimen. The speech of all patients in this retrospective study was reported to be intelligible over the phone. The feasibility of elongating the vascular pedicle when necessary as applied in several cases in this study is another advantage of the fibular graft [9]. A single case report published in 2013 showed uneventful healing and good esthetic outcome of the use of fibular grafts along with a patient specific titanium mesh [10].

Another study used fibular osteocutaneous reconstruction for 27 cases with midfacial defects. Dental implants were placed 3-6 months after the initial surgery and 6 months later the maxillary prosthesis was placed. The study reported a single case of flap failure, while 5 other required intervention to salvage the flap.

### Iliac grafts

Three cases of reconstruction using iliac grafting were found in the selected articles. The use of iliac bone accompanied by the insertion of implants into simultaneously harvested anterolateral thigh flaps was reported in Mueller et al’ s study. The final prosthesis was installed after an average of 13 months postoperatively. Patients wore the prosthesis and were satisfied to an extent although only 3 of the patients wore the prosthesis publicly [11]. Osteomyocutaneous deep circumflex iliac artery perforator was reported to reconstruct the defect caused by midfacial squamous cell carcinoma. The patient showed excellent healing and regain of function and esthetics [12].

### Radial forearm free flap

In reviewing the literature, 40 cases of reconstruction using RFFF as the primary reconstruction graft were found; of which 3 cases were accompanied by ALT soft tissue flap. A case series reported the reconstruction of 23 midfacial defects using osteocutaneous radial forearm free flap (OCRFFF). Recipient site complications ranging from hematoma formation to wound dehiscence was noted in 10 cases. Oronasal fistulas occurred in 3 cases and were locally treated by advancement flaps. While donor site complications occurred in 7 cases with radial bone fracture in 1 case and skin graft loss in 6 others. Although none of the patients received dental rehabilitation, a normal diet was regained except for 2 patients. Smaller defect size understandably showed a lower rate of recipient site complications and showed better esthetic results than larger defects [13]. Another study reported the use of OCRFFF for midfacial reconstruction with /without orbital exenteration. Follow-up lasted for a mean of about 23 months with postoperative moderate deformity reported. Functionally, a normal diet was attained by all patients and understandable speech reported; moreover esthetically all patients returned to their social life postoperatively [14]. Moreno et al reported 2 cases of reconstruction using a RFFF enabling further prosthetic rehabilitation and with satisfactory functional and esthetic outcomes [7]. Another study reported the use of RFFF alone (3 cases) or in conjunction with ALT (1 case) to reconstruct midfacial reconstruction with the insertion of extraoral implants for final prosthetic integration. None of the implants failed and the final prosthesis was integrated after 7-12 months of the microvascular reconstruction [11].

### Scapular flap

Scapular flaps were used in 13 maxillary reconstruction cases in the included articles. A case series published in 1998 reported the use of the scapular grafts to reconstruct midface defects. This was reported in 8 cases; 7 carcinomas and 1 hemangioma. 4 out of the 8 cases required a second procedure due to flap loss or volume correction [15]. The use of a scapular flap with a different vascular pedicle (thoracodorsal artery perforator) has been reported to reconstruct midfacial defects with healing and radiographic evidence of graft consolidation 8 months later. The second case showed fat necrosis at the anterior portion of the graft requiring a secondary surgery to correct the defect but later healing was uneventful [16]. A single case reconstructed with a scapular tip with a composite serratus anterior flap was reported with the graft maintaining viability at the end of the follow-up period and patient reporting satisfactory results [7].

### Calvarial bone graft

A retrospective study of 6 patients reported the reconstruction of the midface using calvarial bone grafts along with a temporoparietal local vascularized graft. The follow-up period extended to a range of 3-36 months with 1 patient complaining of a moderate facial deformity postoperatively due TPF loss and requiring a revision surgery. The authors concluded that the use of calvarial bone grafts in this manner should only be confined to small defects not necessitating further soft tissue grafting [17]. Another retrospective study reported the use of calvarial bone grafts along with a rectus abdominus flap for midfacial reconstruction. Although not all the treated patients received prosthetic rehabilitation that did not affect the functional final reported outcome. It was reported as satisfactory and mastication was reported to be unaffected [18].

### Temporoparietal osteofascial flap

A case series studied the use of temporoparietal osteofascial flap in maxillofacial reconstruction including 2 cases of midface reconstruction. Both cases were reported to show good uneventful healing and successful results along the follow-up periods which lasted 24 and 30 months. The authors concluded that the TOF has the obvious advantage of the absence of a second surgical donor site and so less morbidity. On the other hand, alopecia and dural tears have been reported so extreme care intraoperatively is essential [19].

### Computer guided procedures

The rapidly evolving advancements in the scope of computer software has allowed for the introduction of virtual surgical planning techniques and the fabrication of patient specific implants and/or stereolithographic models.

A single case of the use of a specifically fabricated titanium mesh in conjunction with a latissimus dorsi flap was reported. Mesh exposure was reported requiring secondary surgical intervention with another soft tissue graft. The latissimus dorsi flap provides an abundance of soft tissue which was advantageous in this case to cover the titanium prosthesis. The same amount of soft tissue from another donor site would be accompanied by significant donor-site morbidity [10].

Another case report of a single case with a secondary midfacial depression was treated using a computer guided prefabricated MEDPOR implant. The preoperative CT scans of the patient were used to make a model of the patient’s facial skeleton. The model was then used intraoperatively to adapt the MEDPOR implant. After 9 months of follow-up, both the patient and clinician reported good esthetics and function. The use of MEDPOR in this case avoided a secondary surgical site with its morbidity. On the other hand, the main disadvantage of MEDPOR is the risk of infection and the added cost of the implant [20].

In 2013 a published case report introduced the use of a patient specific implant along with a scapular osteomyocutaneous flap. The patient had an ablative surgery earlier and the CTs were imported into the computer software where a virtual surgery was performed by designing the components of the defect to be reconstructed with the scapular graft (palate and alveolus). A patient specific titanium implant was also designed by mirror imaging the normal side to obtain a structure of the midface as close to normal as possible. The zygomatic and orbital components of the defect were reconstructed using the PSI. Intraoperatively the scapular flap was harvested and microvascular anastomosis achieved by using the facial artery and vein as recipient vessels. Proper insertion and fixation of the device were detected intraoperatively using intraoperative navigation systems. The myocutaneous component of the flap was used to better support the soft tissue of the cheek. Healing was uneventful with good epithelialization of the recipient mucosa and good contour and symmetry. Later on due to the poor vascularity of the radiated recipient bed, as reported by the authors, part of the titanium implant was exposed at the lower eyelid necessitating a secondary rotation cheek flap to cover it. Three months postoperatively complete healing and epithelialization was reported. Although this is a costy technique; it provides superior esthetics and requiring less graft harvesting [21].

## Discussion

Maxillectomy defects have been reported to be treated by prosthetic appliances; obturators; as early as the 1950s aiming to provide adequate esthetics and function after surgical ablation. The advantages of the obturator at that time was to restore functions, reduce bleeding and maintain a clean wound. Obturators have been reported to restore the drinking ability of the patient by closing the oro-antral/nasal caused by the resection [22, 23]. On the other hand, reports of poor masticatory function and improper drinking with an obturator which was attributed to the large initial maxillectomy defect must be taken into consideration [1]. Drawbacks of obturators include leakage, constant need for proper cleaning to maintain hygiene and constant modification of the prosthesis [2]. A higher tendency of improper nasalance; whether hyponasality or hypernasality; in patients receiving obturators post-maxillectomy is also an issue to be considered [24]. Poorer swallowing ability was also reported in patients rehabilitated with an obturator; especially in cases of a larger horizontal defect such as extensive palatal defects (Okay Class III) [7].

Complications of obturators were overcomed with the introduction of microvascular techniques which has become the internationally accepted method of maxillary/midface reconstruction. Donor sites have varied according to the size and type of the defect with pros and cons to each of the reported donor sites in literature.

Fibular grafts are a common option in cases requiring a bony component in the graft. The use of osteocutaneous flaps in midface defects has been reported to be highly reliable and providing acceptable esthetics in cases other than those requiring restoration of bony parts of the orbit. Authors commented that the use of an osteocutaneous fibular graft for midfacial reconstruction is beneficial especially in the freely movable soft tissue component which is not the case in other composite grafts [9]. It has also been reported to successfully provide a platform for the future insertion of dental implants and prosthesis placement although not following the normal anatomy [25]. The use of an osteomyocutaneous fibular flap was also applied in several studies to reconstruct oncologic defects with esthetically and functionally acceptable results to both patients and surgeons [8, 24]. On the other hand, the inadequate alveolar height in cases of mandibular reconstruction has been overcomed by applying the “double-barrel” technique to enable future prosthetic rehabilitation [26, 27]; although this caused a deficiency in bone length [28]. No reports of the application of this technique in maxillary/midface reconstruction were encountered in the literature search.

Radial forearm free flap is another commonly used option. It may supply a wide variety of tissue components; skin, muscle and/or bone according to the defect characteristics and clinician’s preference. Flap harvesting is quite a simple technique with a thin, long reliable pedicle. Osseocutaneous radial forearm grafts have been used to reconstruct midface defects and restoring the infraorbital rim using the bony component of the graft. High success rates in terms of graft incorporation; function restoration (speech and oronasal separation) and esthetic outcomes (patient accepting social interaction) have been reported [14]. Moreover; the presence of a second donor site to harvest the split thickness skin graft increases morbidity. The use of a local full thickness skin graft from the incision already needed to dissect the vascular pedicle of the graft was reported to overcome the necessity of a 2^nd^ donor site. Authors of this trial reported uneventful healing in all 29 cases included with only a single case of seroma formation which was treated locally and eventually healed as well [29]. The use of a narrower and longer skin paddle was introduced allowing easy primary closure of the defect site with satisfactory results [30].

Scapular flaps have also been discussed vastly in the literature with several different applications with/without other tissue grafts. Combination with the latissimus dorsi muscle and harvesting a part of the scapular bone has been reported to provide sufficient bone and soft tissue components to reconstruct maxillectomy defects. Some authors considered it to be the first reconstructive option in cases of maxillectomy with orbital exenteration defects [31] while others noted that the use of the TDA provides a more reliable vascular pedicle with a lower risk of kinking and thrombus formation and the bony component when needed may be sufficient for dental implant placement [16]. Moreover primary closure of the donor site and the longer pedicle are the main advantages of this technique. The anatomical similarity of the scapular tip to that of the palate makes its use for palatal defect reconstruction preferable [32]. The advantages of the scapular flap are; the presence of bony, muscle and skin tissue with proportionate amounts, the long large-caliber donor vessel and the morphological similarity with the maxillary structures [33].

Iliac grafts: the iliac region may provide bone with or without accompanying soft tissue components for maxillofacial reconstruction. The outstanding advantage of iliac grafts is the abundant bone stock available with its vascular pedicle. The cutaneous tissue of the groin has the advantage of being hairless and so mimicking the recipient area but the skin tone unfortunately does not match that of the face. The hairless nature of the groin skin is advantageous although the non-matching skin tone may cause an esthetic concern [12].

Despite the obvious advantage of autogenous tissue grafting, reconstruction of the maxilla/midface has higher morbidity rates and is a complex procedure. Prolonged surgical time complications, donor site morbidity, technique sensitivity and expensive equipment complicate the use of microvascular surgery. With the introduction of computer-guided surgeries, preoperative surgical planning and fabrication of patient specific implants were reported and aimed at overcoming such drawbacks [8, 21].

The technique of computer-assisted planning and mirror-imaging of the same patient’s normal side was used by Mertens et al to fabricate a patient specific titanium implant (PSI) to act as a scaffold for an osteomyocutaneous scapular flap to reconstruct an oncologic midface defect. Postoperative CT was ordered at the end of the 4 month followup period to enable the fabrication of implant-retained rehabilitation was used. The authors considered it to be a promising technique combining advantages of several reconstructive techniques yet no further details on the postoperative period have been provided [26]. The main obstacle to generalize the use of computer guided and navigation modalities is their cost [34]. Preoperative planning of both donor and recipient sites is also done to attain precise bony harvests to fit the preplanned bed. This may be performed by printing models of the recipient and donor sites and shaping the donor graft accordingly preoperatively [8]. Virtual planning may greatly reduce human error by mirror-duplication of the contralateral side in cases of hemifacial defects and so attaining better facial symmetry than that planned arbitrarily [35]. Although several computer applications have been introduced; the use of these applications remains quite complicated and requires extensive training. These techniques have undoubtedly made the intraoperative surgical steps easier and their outcome expectable. Intraoperative surgical time previously wasted in planning, adaptation and even evaluating surgical steps has been saved by finishing these steps preoperatively. The reduced intraoperative time reduce surgical cost and complications.

In conclusion, the articles included in this review were chosen to target methods used to overcome facial asymmetry/deformity caused by pathologic defects. The lack of controlled/ RCTs and a fixed postoperative evaluation scale made the comparison of the results impossible. It was noted however that outcomes were directly affected by the extent of the defect with larger defects usually accompanied by poorer outcomes. The computer-guided novel techniques are yet to be researched to allow for further assessment but the show great potential for esthetically satisfactory simple midface reconstruction. The risk of bias in all assessed articles was substantially high making the proper evaluation of the reported techniques impossible. Outcomes in the selected articles were vague reports of patients’ satisfaction as regards function and esthetics. Properly arranged RCTs with fixed postoperative assessment measures are highly recommended to enable further analysis of the results to come up with guidelines for midface/maxillary reconstruction.
